# The potential clinical impact of cell type‐specific genetic regulation: Crohn's disease

**DOI:** 10.1002/ctm2.1474

**Published:** 2023-11-20

**Authors:** Rika Tyebally, Angli Xue, Joseph E. Powell

**Affiliations:** ^1^ Translational Genomics Garvan Institute of Medical Research, Darlinghurst Sydney New South Wales Australia; ^2^ UNSW Cellular Genomics Futures Institute University of New South Wales, Kingston Sydney New South Wales Australia; ^3^ School of Biomedical Sciences University of New South Wales Sydney New South Wales Australia

**Keywords:** Crohn's disease, gene networks, genomics, single cell

## Abstract

Complex diseases are heterogenous due to variation in their genetic and environmental underpinnings, leading to varied treatment responses. Genome‐wide association studies (GWAS) integrated with single‐cell expression quantitative trait loci analyses (eQTL) can pinpoint cell‐type specific candidate disease‐relevant genes and pathways. This knowledge can be applied to patient stratification and novel therapeutic target identification. Here, we describe the translational potential of cell‐type specific genetic regulation, using Crohn's disease as an example.

## 1 INTRODUCTION

Complex diseases, such as cardiovascular disease, autoimmune disease and neurodegenerative disease, are common in the population and underpinned by many genetic and environmental factors. They present a spectrum of pathophysiology, due to inherent genetic and environmental variation between individuals and the complexity of cellular and molecular systems. Consequently, this variation extends to the treatment responses in patients.^1^ Current treatments are often not curative, can have low efficacy, and are often administered in a *one‐size‐fits‐all* approach. To address these problems, personalised medicine has become a key aim of biomedical research. Personalised medicine involves developing biomarkers or clinic‐based tests that can predict disease progression or treatment response for an individual patient. If applied successfully, personalised medicine will lead to efficient patient stratification, improved patient outcomes, and ultimately, economic benefits for healthcare.

Genome‐wide association studies (GWAS) for complex diseases have identified thousands of genomic loci associated with disease.^2^ Many of these variants are located in noncoding regions of the genome and impact disease risk through changes in genome regulation—such as changing the expression levels of genes.^3^ Functional interpretation of GWAS results requires follow‐up analyses, including statistical and experimental validation.^4^ An important component of this functional interpretation is the use of expression quantitative trait loci (eQTL) analyses, which identify associations between genetic variants (typically Single Nucleotide Polymorphisms, SNPs) and gene expression.^5^ Based on the hypothesis that altered gene expression is a mediator of disease risk, GWAS and eQTL results can be integrated to identify candidate “causal genes.”^6^, ^7^

Large‐scale eQTL analyses on single‐cell RNA‐seq data (instead of traditional bulk‐RNA‐seq data) have shown that the effect of genetic variants on gene expression is highly cell type‐specific^8^; that is, a genetic variant will influence the expression of a gene in one cell type, but the same variant will not have this effect in another cell type.^9^, ^10^ Moreover, eQTLs are also specific to cell states and contexts, for example, only observed in cells responding to a pathogen.^11^, ^12^, ^13^ In summary, the effect of individual genotypes on molecular phenotypes such as gene expression is nuanced, but technology and efficient experimental designs are revealing how disease risk loci functionally act at the resolution of individual cells. This area of research is used to facilitate fine‐mapping disease‐associated variants, identify causal genes and corresponding cell types, and provide an avenue to stratify patients based on cell type‐specific genetic effects.

Here, we describe the translational potential of identifying how disease risk loci have cell type‐specific effects on genome regulation. Determining the impact of cell type‐specific disease risk loci can be facilitated through machine‐learning or artificial intelligence approaches that link genetic variation to molecular phenotypes and the subsequent effects on gene regulatory networks. We see two clear avenues for short‐ and long‐term clinical translation originating with identifying these disease‐ and cell‐specific gene regulatory networks. Firstly, the development of companion tests for predicting patient response to current therapies can be achieved by identifying how genetic differences between patients affect the molecular levels of a drug's target; secondly, by identifying novel targets, particularly those amenable to RNA‐based therapeutics. While developing an effective new therapy has many hurdles, the approaches for target identification described above are of particular value as they can link the genetic effects of a disease to causal pathways at the level of individual cells. Identifying strong evidence for causal mechanisms of disease underlying most known genetic loci is also achievable using careful experimental designs with high scalability. In this perspective, we use Crohn's disease to illustrate how these approaches may yield valuable translational outcomes, although the concepts described below apply to most diseases.

### 1.1 Treatment for Crohn's disease will be improved through patient stratification based on genetics

Crohn's disease is a subtype of inflammatory bowel disease (IBD), a group of complex diseases characterised by chronic inflammation in the gastrointestinal tract. IBD tends to occur in genetically susceptible individuals and is linked to environmental factors such as diet and lifestyle. The key cellular and molecular traits in Crohn's disease include dysregulated immune signaling in the gastrointestinal tract, a compromised gut epithelium, and altered microbiome.^14^, ^15^ Treatment involves steroids for mild‐moderate Crohn's disease and immunosuppressants and biologics (e.g., anti‐TNFα antibodies, anti‐integrin antibodies, IL‐23/‐12 antagonists) for moderate to severe disease.^16^ Importantly, treatment is either inefficient in a fraction of patients, loses efficacy over time or treatment dependence arises.^16^, ^17^ There is a clear need for approaches to stratify patients to improve treatment response and develop new effective therapies.^17^, ^18^, ^19^

Large IBD/Crohn's disease cohorts with clinical history and genotype information have been curated and enabled efforts to identify biomarkers for predicting treatment response and prognosis of the disease over time. A high‐level overview of these, which are in development or clinical trial stages, is reviewed in Verstockt et al.^19^ In summary, stratification based on transcriptomic, proteomic and microbiomic markers is promising, but no such stratification methods currently exist. Further, many challenges such as cost, reproducibility, interpretability, and time to commercialization, remain to be addressed.^19^

### 1.2 Genetic regulation of Crohn's disease drug target genes

Crohn's disease risk has a significant genetic component, with 75%−79% of the population risk estimated to be due to genetic variation.^20^, ^21^, ^22^ These high estimates have motivated IBD genome‐wide association studies over the last 17 years,^23^, ^24^, ^25^, ^26^, ^27^, ^28^, ^29^ which have collectively identified 320 loci associated with IBD,^20^ and contributed to the identification of genes that regulate the cellular processes that underlie Crohn's disease (e.g., *NOD2* in innate immunity and *ATG16L1* in autophagy). A key example is the *IL23* locus, which led to the successful development of the IL23/12 p40 antagonist ustekinumab for Crohn's disease.^30^, ^31^ However, many other associated loci continue to require characterisation.

OneK1K, the largest single‐cell eQTL study conducted to date, generated genotype data from 1000 individuals along with single‐cell RNA‐seq on ∼1000 peripheral blood mononuclear cells (PBMCs) per donor.^9^ Data analysis identified how genetic loci are associated with disease‐relevant immune signaling genes in a cell type‐specific manner (Table 1). These genes include those targeted by Crohn's disease therapies, such as TNFα, integrin adhesion molecule α4β7, IL‐23/‐12, and genes that belong in their signaling pathways.^9^ These data provide empirical evidence that the genetic profiles of a patient influence the expression levels of drug targets and consequently could be used to predict patient response. This is supported by evidence that variation in response to anti‐TNFα agents has been shown to be due to drug concentration requirements and recommendations of patient‐specific dosage concentrations.^18^

**TABLE 1:** Impact of genetic variation on the expression levels of Crohn's disease therapies in relevant cell sub‐types.^9^ *The fold change is an average ratio of the transcript molecules per cell between patients with homozygous Allele1 and homozygous Allele2.

Mechanism of action	Drug	Gene(s)	Cell types expressing gene	SNP(s)	Allele 1	Allele 2	Fold change*
TNFα antagonist	Adalimumab	*TNF*	macrophages, monocytes, T‐ & NK‐cells	rs3138155	T	A	6.4x
	Golimumab	*TNF*	macrophages, monocytes, T‐ & NK‐cells	rs3138155	T	A	6.4x
	Infliximab	*TNF*	macrophages, monocytes, T‐ & NK‐cells	rs3138155	T	A	6.4x
Integrin α4β7 antagonist	Vedolizumab	*ITGA4*	Lymphocytes	rs13033467	A	G	7.2x
IL‐23/12 antagonist	Ustekinumab	*IL12A, IL12B*, *IL23A*	Dendritic cells, macrophages, B‐cells, T‐cells	rs17885551 rs845381	T G	C A	7.8x 6.4x
	Risankizumab	*IL23A*	T‐cells, NK, Monocytes, and B‐cells (IL23 only)	rs845381	G	A	6.4x
Sphingosine‐1‐phosphate (S1P) receptor agonist	Ozanimod	*S1PR1, S1PR5*	NK, DC, Monocytes, T‐cells, B‐cells	rs10418489 rs28580742 rs11166573	G T G	A C A	6.6x 10.4x 7.4x
JAK inhibitor	Tofacitinib	*JAK1, JAK3*	B and T lymphocytes	rs73001498	G	A	7.2x
	Upadacitinib	*JAK1*	B and T lymphocytes and NK cells	rs73001498	G	A	7.2x

### 1.3 Impact of cell type‐specific genetic effects on gene regulatory networks

The translational impact of linking disease risk loci to cell type‐specific regulatory effects is strengthened by incorporating gene regulatory networks^32^ – essentially models of regulatory relationships of genes, resolved at a cell level. As genetic loci impact the expression levels of a gene, they also impact gene and protein pathways. It has been proposed that creating gene regulatory networks of individual cell types and applying eQTL knowledge to these networks will facilitate understanding the effect of eQTLs on gene regulation at the transcript level.^32^ This would provide a holistic view of the effect of genetic variation on gene expression, as it expands from individual genes to pathways. Knowing how genetic effects work across pathways will help resolve how a patient's genetic profile contributes to disease mechanisms and treatment response, as well as help identify genomic vulnerabilities for new therapies.

Apart from transcriptional regulation, other features such as metabolic reactions, cell signaling and protein‐protein interaction can also be modelled into a network. Such *multi‐modal* networks are more likely to recapitulate biological complexity but have required the development of additional methods to identify the genomic relationships accurately. For example, several methods have been published that incorporate chromatin accessibility with gene expression data to create gene regulatory networks.^33^, ^34^ Currently, successful outcomes from single‐cell multi‐modal gene regulatory networks have been based on transcriptome and chromatin accessibility data.^35^ However, integration across additional ‐omic levels (i.e., proteomic and metabolomic) is on the horizon.^36^

### 1.4 Framework to identify cell type‐specific drug target landscapes and disease‐relevant pathways

**FIGURE 1:**
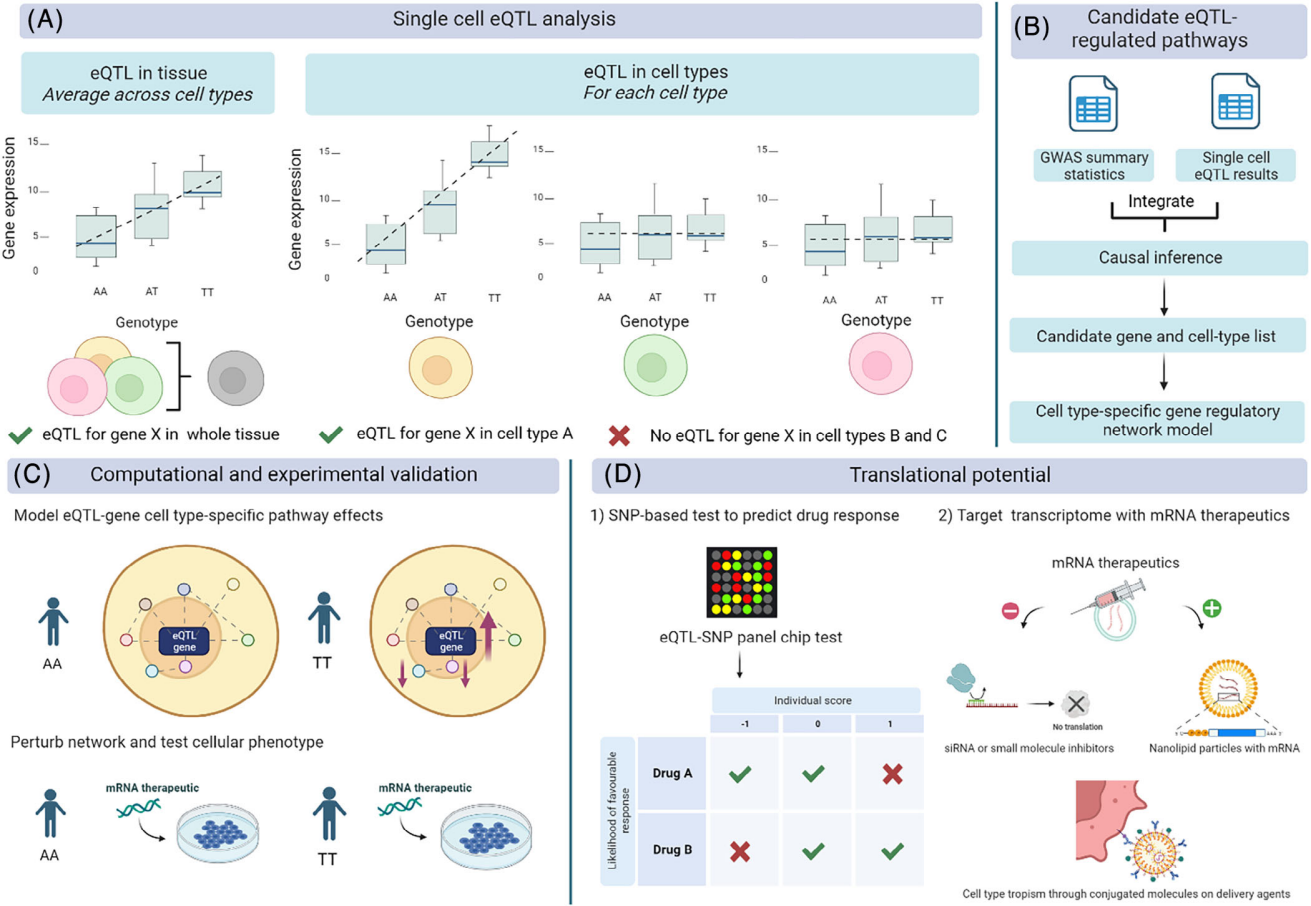
The translational and predictive potential of cell type‐specific genetic regulation. (A) Single cell RNA‐seq eQTL analyses can resolve averaged genetic effects detected in bulk RNA‐seq eQTL analyses (shown); or, conversely, may detect cell type‐specific signals otherwise undetected due to the averaging effect in bulk RNA‐seq eQTL analyses. (**B**) Workflow for identifying candidate eQTL‐regulated genes (eGenes) for further investigation. Causal inference methods integrate GWAS and eQTL summary statistics to pinpoint disease genes. These genes can be further investigated to create a model gene regulatory network for a specific cell type. (**C**) Computational simulation methods can test the effect of quantitative variation of candidate eGenes in a regulatory network. These effects can be tested in vitro, with a disease‐phenotype molecular or cellular readout. (**D**) Future directions for cell type‐specific genetic regulation profiling include (1) development of SNP‐genotyping clinical tests for patient stratification, and (2) identifying novel therapeutic targets amenable to RNA therapeutics.

In this Perspective, we propose a framework for inferring the downstream effects of cell type‐specific eQTLs from single‐cell transcriptomic data. Our approach employs statistical tools, literature, online databases and network inference tools (Figure 1).

Candidate disease‐causal genes are hypothesized to impact disease via dosage variation (eQTL regulation). They can be identified using statistical methods, such as Mendelian randomization.^7^ Genes identified through these approaches have evidence of plausible causal association with a disease, where disease risk is mediated via increases or decreases in gene expression. Often many such genes are identified, which is expected given the complexity of the genetic architecture of complex diseases. This long‐list of genes can be filtered, based on known biological functions (for example immune signaling, autophagy, or microbial interactions), and the specificity of the eQTL effect (the genetic variant uniquely affects gene expression in one cell type but not others). Additional information such as known protein interactions, splice variation and protein function can help determine links between candidate genes and established druggable pathways. In doing so, we can simultaneously identify a drug target landscape, and understand the mechanisms of drug response variation in a cell type‐specific manner.^37^

### 1.5 Future directions: Treatment response‐predicting SNP‐based tests and mRNA therapeutics

#### 1.5.1 Treatment response‐predicting SNP‐based tests

We foresee that the concepts outlined here could lead to curating a set of common genetic loci that can be incorporated into a microarray chip for testing in the clinic. This array will represent SNPs known to influence the expression of drug target genes and their signaling pathway counterparts in a way that affects treatment response. This is conceptually similar to polygenic risk scores, where an individual is genotyped, and the occurrence and effect sizes of multiple independent risk‐associated SNPs are aggregated to estimate the genetic susceptibility for a given trait. The strength and novelty of the SNP‐panel for treatment response proposed here lies in its knowledge of the cell‐types involved, and that the risk SNPs have functional characterisation as eQTL‐SNPs.

#### 1.5.2 Small molecule therapeutics

Therapeutic modulation of gene expression can be achieved using drugs that target RNA transcripts. The field of nucleic acid‐based therapeutics is growing rapidly. Many are U.S. Food and Drug Administration (FDA)‐approved or in clinical trials; such as antisense oligonucleotide Nusinersen for spinal muscular atrophy.^38^ There are several mechanisms of action for this class of drugs, including (1) antisense oligo nucleotides that bind to RNA molecules based on Watson‐Crick base pairing; (2) mRNA molecules that are translated by cellular machinery to proteins; (3) siRNA molecules to knockdown target transcripts; and (4) small molecules that target RNA structures which modulate cellular functions.^38^ Of particular interest are methods to modulate gene expression by decreasing and increasing mRNA transcript levels, which may rectify the altered gene expression signatures conferred by risk‐ or treatment response‐associated genotypes. Additionally, methods for delivering these therapeutics include lipid nanoparticles and endosome packaging. Lipid nanoparticle packaging can also be conjugated with antibodies to allow for cell‐type tropism, allowing cell type‐specific targeting of nucleic acid regulatory drugs.^39^

## 2 CONCLUSIONS

In conclusion, the exploration of cell type‐specific genetic regulation presents a step forward in personalised medicine, especially for complex diseases like Crohn's disease. By integrating data from GWAS, eQTL analyses, and single‐cell RNA‐seq, we can now uncover nuanced genetic influences that manifest differently across cell types, ultimately affecting disease progression and treatment response. This approach not only helps in identifying the genetic underpinnings of diseases but also holds the key to understanding the variable efficacy of current therapies at an individual level.

The translational potential of this research is substantial. In the short term, it facilitates the development of predictive models and companion diagnostics that stratify patients based on their unique genetic profiles, potentially guiding more effective treatment regimens. In the long term, it provides a roadmap for identifying novel therapeutic targets and developing new treatments, particularly RNA‐based therapeutics that can modulate gene expression.

However, challenges remain, including the cost, reproducibility, and commercialization of stratification methods, and the complexity of integrating multi‐modal ‐omic data to fully capture biological intricacies. Additionally, while the focus here is on Crohn's disease, the principles and methodologies applied are broadly relevant to other complex diseases, underscoring the universal applicability of this approach in enhancing personalised medicine.

Ultimately, this research paradigm underscores the importance of understanding diseases not just at the molecular or systemic level, but at the resolution of individual cells, considering the interplay of genetics and cell type specificity. This could improve treatment protocols, shifting from a one‐size‐fits‐all approach to truly personalised therapeutic strategies, thereby improving patient outcomes and the overall efficacy of healthcare systems.

## AUTHOR CONTRIBUTIONS

RT, AX, and JEP conceived the idea. RT and JEP wrote the manuscript, with input from AX. All authors reviewed and provided feedback on the manuscript.

## CONFLICT OF INTEREST STATEMENT

The authors declare no competing interests.

## FUNDING INFORMATION

National Health and Medical Research Council (NHMRC), Grant Number: 1175781; The Goodridge Foundation.
